# Opinions and Beliefs About Telemedicine for Emergency Treatment During Ambulance Transportation and for Chronic Care at Home

**DOI:** 10.2196/ijmr.5015

**Published:** 2016-03-30

**Authors:** Alexis Valenzuela Espinoza, Ann De Smedt, Kaat Guldolf, Fenne Vandervorst, Robbert-Jan Van Hooff, Helio Fernandez Tellez, Sara Desmaele, Melissa Cambron, Ives Hubloue, Raf Brouns

**Affiliations:** ^1^ Center for Neurosciences (C4N) Vrije Universiteit Brussel (VUB) Brussels Belgium; ^2^ Department of Public Health Vrije Universiteit Brussel Brussels Belgium; ^3^ Universitair Ziekenhuis Brussel Department of Neurology Brussels Belgium; ^4^ Research group Clinical Pharmacology and Clinical Pharmacy (KFAR) Vrije Universiteit Brussel (VUB) Brussels Belgium; ^5^ Department of Emergency Medicine Universitair Ziekenhuis Brussel Brussels Belgium; ^6^ Research Group on Emergency and Disaster Medicine (ReGEDiM) Vrije Universiteit Brussel (VUB) Brussels Belgium

**Keywords:** telemedicine, stroke, adoption, diagnostic techniques and procedures, emergency care, patient-centered care

## Abstract

**Background:**

Telemedicine is a valid alternative to face-to-face patient care in many areas. However, the opinion of all stakeholders is decisive for successful adoption of this technique, especially as telemedicine expands into novel domains such as emergency teleconsultations during ambulance transportation and chronic care at home.

**Objective:**

We evaluate the viewpoints of the broad public, patients, and professional caregivers in these situations.

**Methods:**

A 10-question survey was developed and obtained via face-to-face interviews of visitors at the Universitair Ziekenhuis Brussel (UZB). The online questionnaire was also distributed among professional caregivers via the intranet of the UZB and among the broad public using social media.

**Results:**

In total, 607 individuals responded to the questionnaire, expressing a positive opinion regarding telemedicine for in-ambulance emergency treatment and for chronic care at home. Privacy issues were not perceived as relevant, and most respondents were ready to participate in future teleconsultations. Lack of telecommunication knowledge (213/566, 37.6%) was the only independent factor associated with rejection of telemedicine at home and respondents via social media (250/607, 41.2%) were less concerned about privacy issues than respondents via face-to-face interviews (visitors, 234/607, 38.6%). The visitors were more positive towards in-ambulance telemedicine and more likely to agree with future participation in teleconsultations than respondents via social media.

**Conclusions:**

The broad public, professional caregivers, and patients reported a positive attitude towards telemedicine for emergency treatment during ambulance transportation and for chronic care at home. These results support further improvement of telemedicine solutions in these domains.

## Introduction

Telemedicine has been shown to be a reliable, sustainable, and cost-effective alternative for face-to-face patient care in many medical domains [1-4]. Yet, the adoption of telemedicine in routine health care has been slow and fragmented since its introduction some 50 years ago [5,6]. Key technological components for telehealth applications have come of age and are readily available at an acceptable cost, but several hurdles still need to be cleared to allow valid results. The cultural barrier, that is, the reluctance of patients and caregivers to adopt novel practices, is often perceived as a major issue [7,8] and requires more research. Opinions held by the general public, professional caregivers, and patients may differ, and a better insight into the potential role of computer illiteracy and demographics is critical for telemedicine to become a part of the everyday medical practice. Furthermore, it is unknown if all stakeholders support the expansion of telemedicine into novel domains such as hyper-acute treatment during emergency ambulance transportation [9,10] and chronic care at the patient’s home [11].

This study characterizes and compares the viewpoints of the general public, health care professionals, and stroke patients on telemedicine for emergency treatment during ambulance transportation and for chronic care at home.

## Methods

### Survey

We designed a concise 10-question survey, which typically took less than 5 minutes to complete. The questionnaire was available in Dutch and French. In-ambulance telemedicine support for patients with suspicion of acute stroke (telestroke) was used as a showcase for emergency telemedicine [9]. The survey was available via an Internet website and contained questions related to preferred language, demographics, history of stroke, and knowledge of computer systems for telecommunication. Using 5-point Likert scales [12], we questioned the respondents’ opinions about in-ambulance telestroke, telemedicine at home, protection of privacy and identity, and willingness to participate in future telemedicine consultations (see Table 1). A composite score reflecting a respondent’s overall attitude towards telemedicine was computed by summation of all individual responses on the four Likert-scale questions. The answers “Strongly disagree,” “Disagree,” “Neutral,” “Agree,” and “Strongly agree” were attributed 1, 2, 3, 4, and 5 points, respectively.

### Study Population

The survey was conducted via face-to-face interviews of visitors at the Universitair Ziekenhuis Brussel (UZB) on World Stroke Day (October 29, 2014) and was available online for 1 month following this day. Visitors who participated in the face-to-face interviews had access to a prototype of an in-ambulance telestroke system at the site and additional information at their request. The online questionnaire was distributed among professional caregivers via the UZB Intranet and among the general public using social media (email, Facebook). We identified the type of respondents based on Internet protocol addresses. Specific addresses correlated with the UZB Intranet, which is accessible only for UZB employees (referred to as professionals), and with the computers used for face-to-face interviews with UZB visitors on World Stroke Day (referred to as visitors). All other addresses were associated with respondents who accessed the survey via distribution through social media (referred to as social media). Only the results of respondents aged 18 years and older who provided at least one answer were taken into account. The data collection was anonymous, and no personally identifiable data related to individuals were collected.

**Table 1:** The 10-question survey.

Questions	Answers
Q1. Preferred language:	Dutch or French
Q2. What is your age?	Numeric input
Q3. What is your gender?	Female or Male
Q4. Did you suffer a stroke in the past?	Yes, I don’t know, or No
Q5. Do you use computer systems for telecommunication, for instance, Skype?	Yes, I don’t know, or No
Q6. In case of a stroke, I would like to receive support via telemedicine during transportation by ambulance to the hospital:	Strongly disagree, Disagree, Neutral, Agree, or Strongly agree
Q7. I find the use of telemedicine for patient care at home useful:	Strongly disagree, Disagree, Neutral, Agree, or Strongly agree
Q8. I am confident that my privacy and identity would be protected during telemedicine consultations:	Strongly disagree, Disagree, Neutral, Agree, or Strongly agree
Q9. I would like to participate in telemedicine consultations in the future:	Strongly disagree, Disagree, Neutral, Agree, or Strongly agree
Q10. Comments and suggestions:	Free text

### Statistical Analysis

Univariate testing was performed to identify associations between possible confounding factors (language preference, age, gender, history of stroke, knowledge of computer systems for telecommunication) and the four Likert-scale questions about telemedicine. Pearson’s chi-square test or Fisher’s exact test were used for categorical variables, as appropriate. For continuous variables, the Spearman correlation, the Mann-Whitney U test, or the Kruskal Wallis test of variance were applied. Multivariate regression analysis by a forward stepwise method was performed with entry and removal criteria of 0.05 and 0.10, respectively, including all variables <0.05 in univariate analysis. Shift analysis of the Likert scale score was assessed by the van Elteren Cochran-Mantel-Hanszel test with adjustment for variables with significant association in univariate analysis [13]. The internal consistency of the survey was assessed by Cronbach alpha. Statistical computations were performed with the SPSS software package version 22.0, except for evaluation of the Likert-scale shift, which was carried out in Stata version 13.

## Results

### Study Population

In total, 642 respondents accessed the Web-based survey, of whom 607 were aged ≥18 years and provided at least one answer. We received 577 answers (95.1%) in the first 5 days after launch of the survey; 536 respondents preferred to complete the survey in Dutch (88.3%). The respondents’ median age was 47 years (interquartile range [IQR] 29-57 years) and 388 respondents were female (63.9%). Nineteen respondents (3.1%) reported a previous stroke, and 8 respondents indicated that they did not know whether they had suffered from a stroke (1.3%). Patients with a (possible) history of stroke were significantly older and more often male than respondents without history of stroke (*P*<.001 for both).

Of 213 respondents (37.6%), we inferred that they lack knowledge of computer systems for telecommunication, as 209 respondents indicated that they did not have this knowledge and 4 respondents did not know whether they had this knowledge.

Of the 607 respondents, we identified 123 as professional (20.3%), 234 as visitor (38.6%), and 250 as social media (41.2%). Table 2 summarizes the characteristics of the three respondent types. Visitors less frequently preferred the Dutch language than professionals or respondents via social media (*P*<.001). Visitors were more frequently male than professionals (*P*=.001), but there was no significant gender difference between visitors and respondents via social media. Visitors were older than professionals and respondents via social media (*P*<.001 for both), and more often reported previous stroke than professionals (*P=*.018) but not more than respondents via social media (*P=*.104). Visitors more frequently had no knowledge of computer systems for telecommunication than professionals (*P=*.019) and respondents via social media (*P*<.001). There were no significant differences in baseline characteristics between professionals and respondents via social media, except for more female respondents in the subgroup of professionals (*P=*.024).

### In-ambulance Telestroke

**Figure 1:**
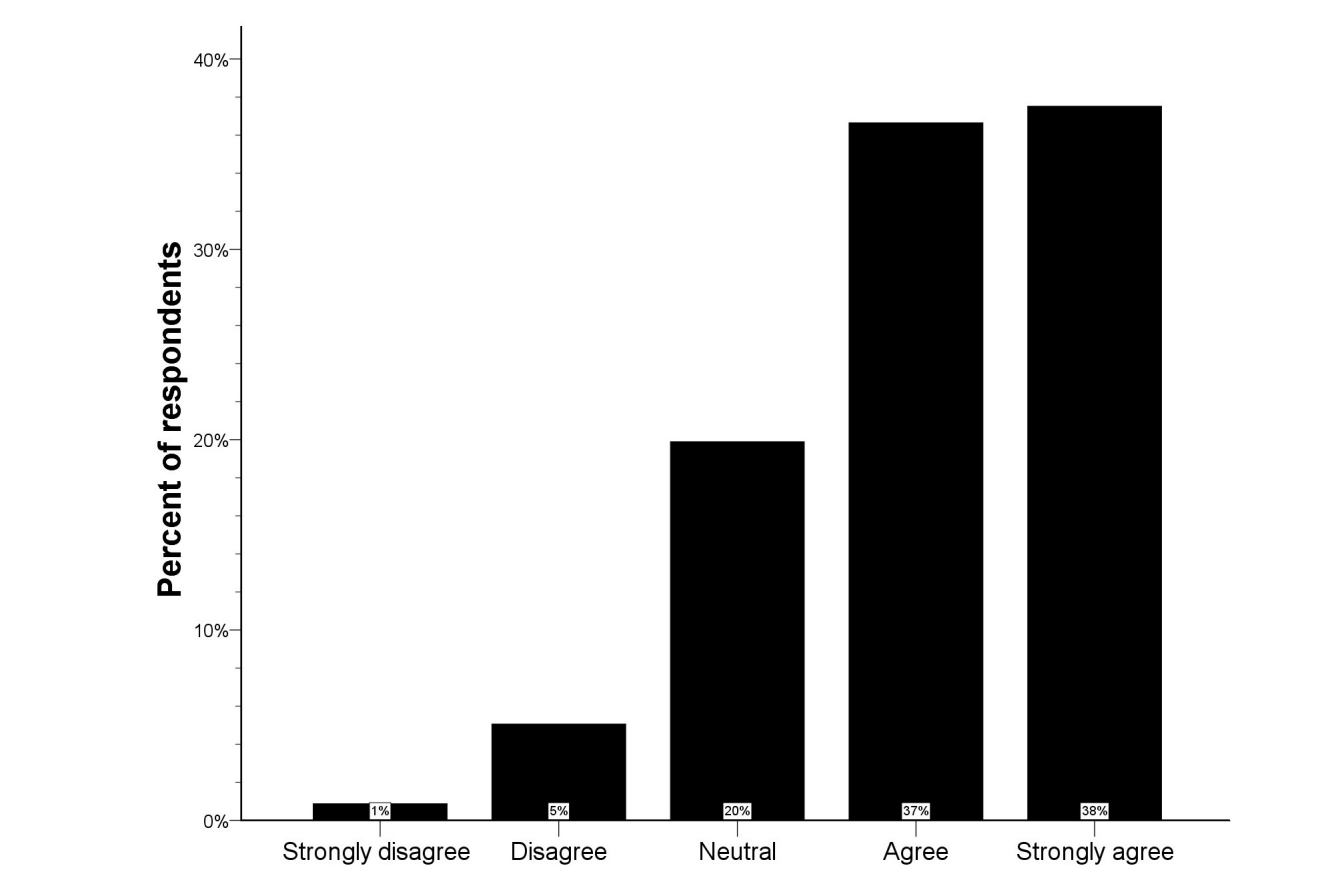
Distribution of the Likert scale for in-ambulance telestroke in the total study population.

**Figure 2:**
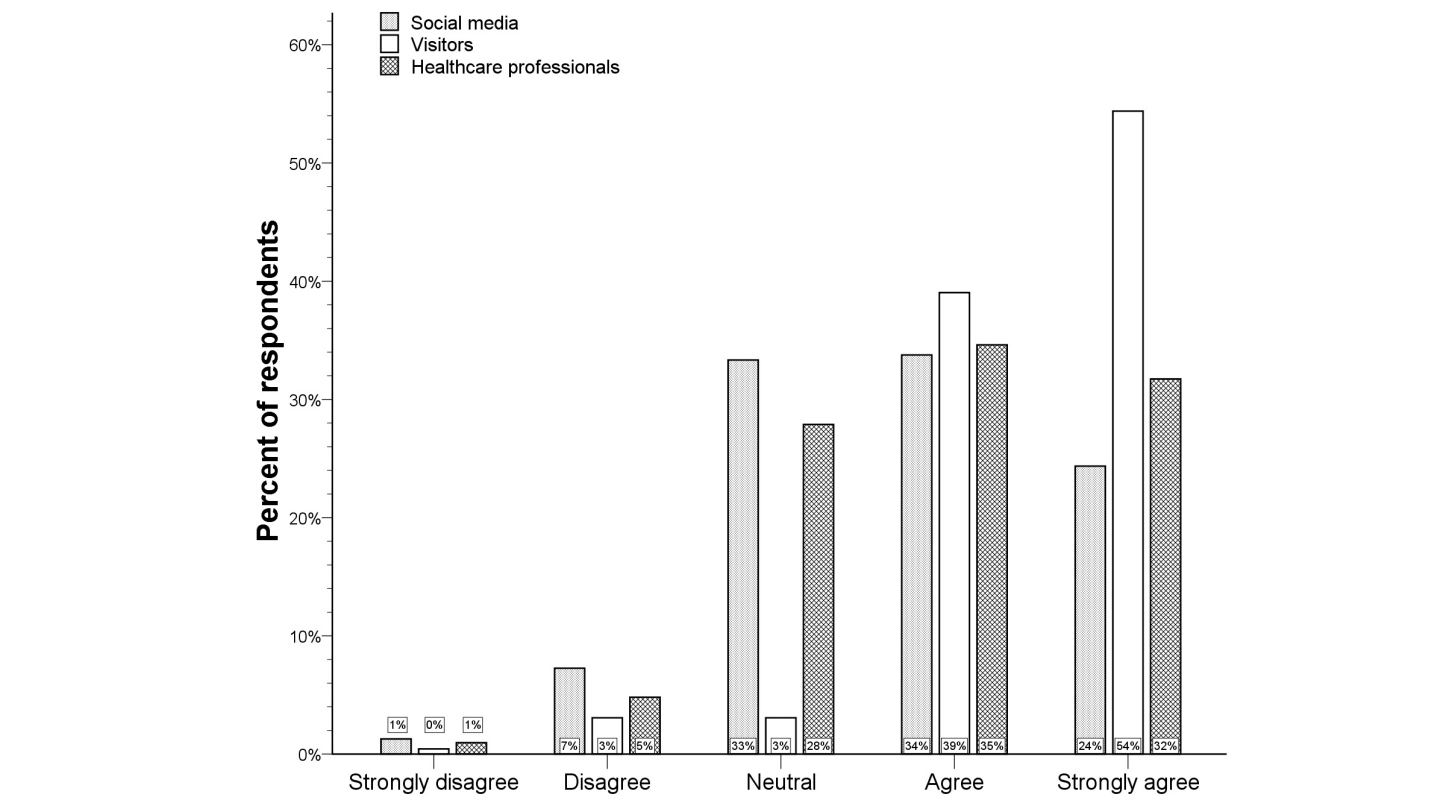
Distribution of the Likert scale for in-ambulance telestroke per respondent type.

**Figure 3:**
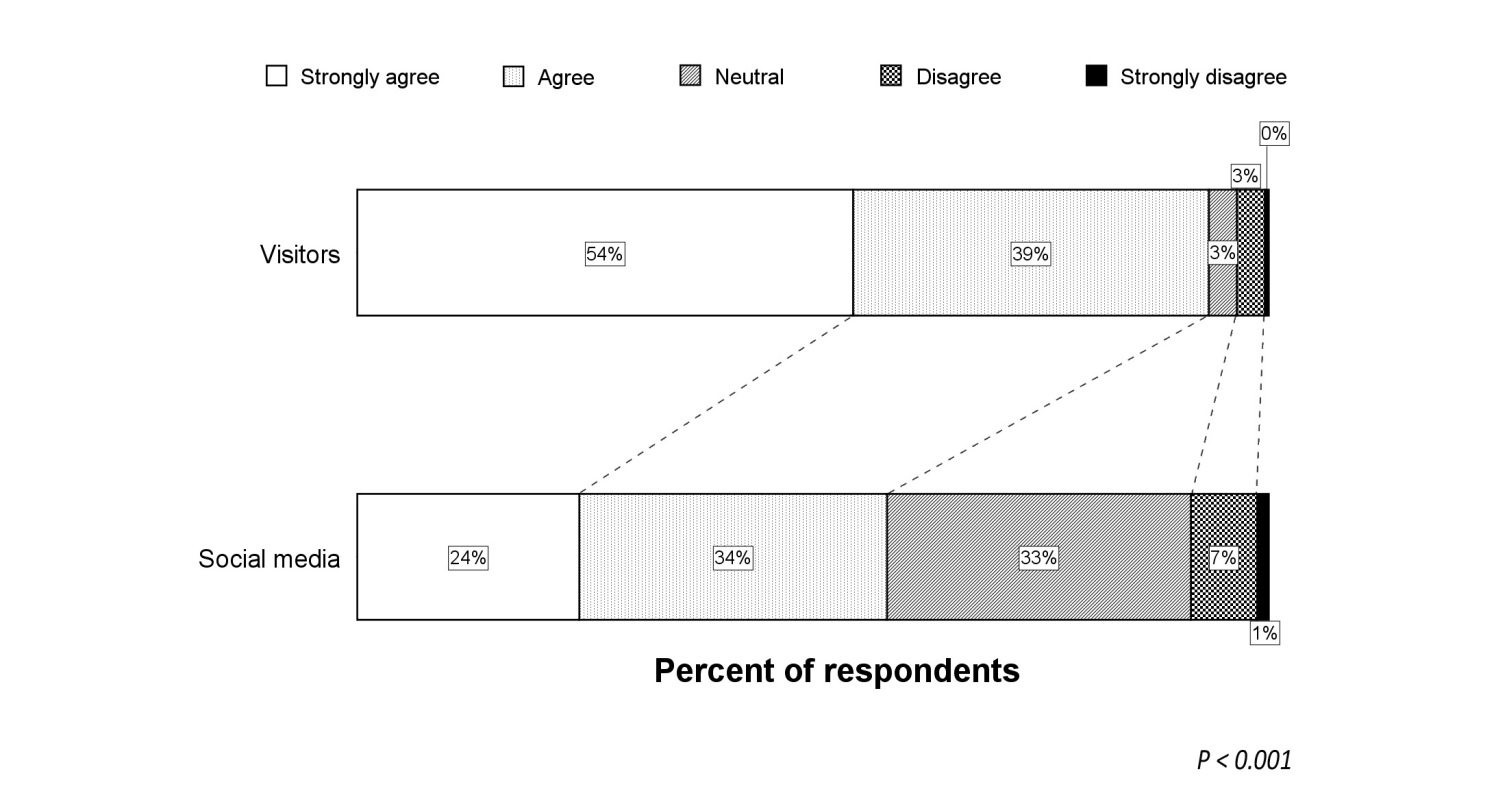
Shift analysis of the responses from visitors compared to social media concerning in-ambulance telestroke.

The Likert scale distribution for the question regarding in-ambulance telestroke for the total study population is illustrated by Figure 1. Very few respondents (6.0%) did not wish to receive in-ambulance telestroke (median score 4, IQR 3-5). Univariate analysis showed higher Likert scale scores for French-speaking respondents and older respondents (*P*<.001 for both). Visitors more frequently agreed and strongly agreed with in-ambulance telestroke than respondents via social media or professionals (*P*<.001 for both) (see Figure 2). Logistic regression analysis identified the respondent type as an independent predictor for acceptance (ie, “Strongly agree” or “Agree” vs “Disagree” or “Strongly disagree”) of in-ambulance telestroke (OR 3.9, 95% CI 1.7-9.1; *P=*.02). Controlling for preferred language and age, the distribution of the Likert scale responses was significantly more favorable in visitors, as compared to respondents via social media (*P=*.001) (see Figure 3).

**Table 2:** Baseline characteristics of the three respondent types (N=607).

Parameter		Social media (n=250, 41.2%)	Visitor (n=234, 38.6%)	Professional (n=123, 20.3%)	*P* value
Dutch language (n, %)^a^		249 (99.6)	167 (71.4)	123 (97.6)	<.001
Female gender (n, %)^b^		160 (64.0)	135 (57.7)	93 (75.6)	.004
Age (median, IQR) ^c^		40 (26-53)	54 (40-65)	45 (29-53)	<.001
**Previous stroke (n, %)** ^a^					.021
	No	243 (97.2)	219 (93.6)	118 (95.9)	
	Don’t know	2 (0.8)	2 (0.9)	4 (3.3)	
	Yes	5 (2.0)	13 (5.6)	1 (0.8)	
**Knowledge of telecommunication (n, %)** ^a^					<.001
	No	59 (25.1)	112 (49.6)	38 (36.2)	
	Don’t know	3 (1.3)	0 (0.0)	1 (1.0	
	Yes	173 (73.6)	114 (50.4)	66 (62.9)	

^a^Fisher’s test.

^b^Chi-square test.

^c^Kruskal Wallis test of variance.

### Telemedicine at Home

**Figure 4:**
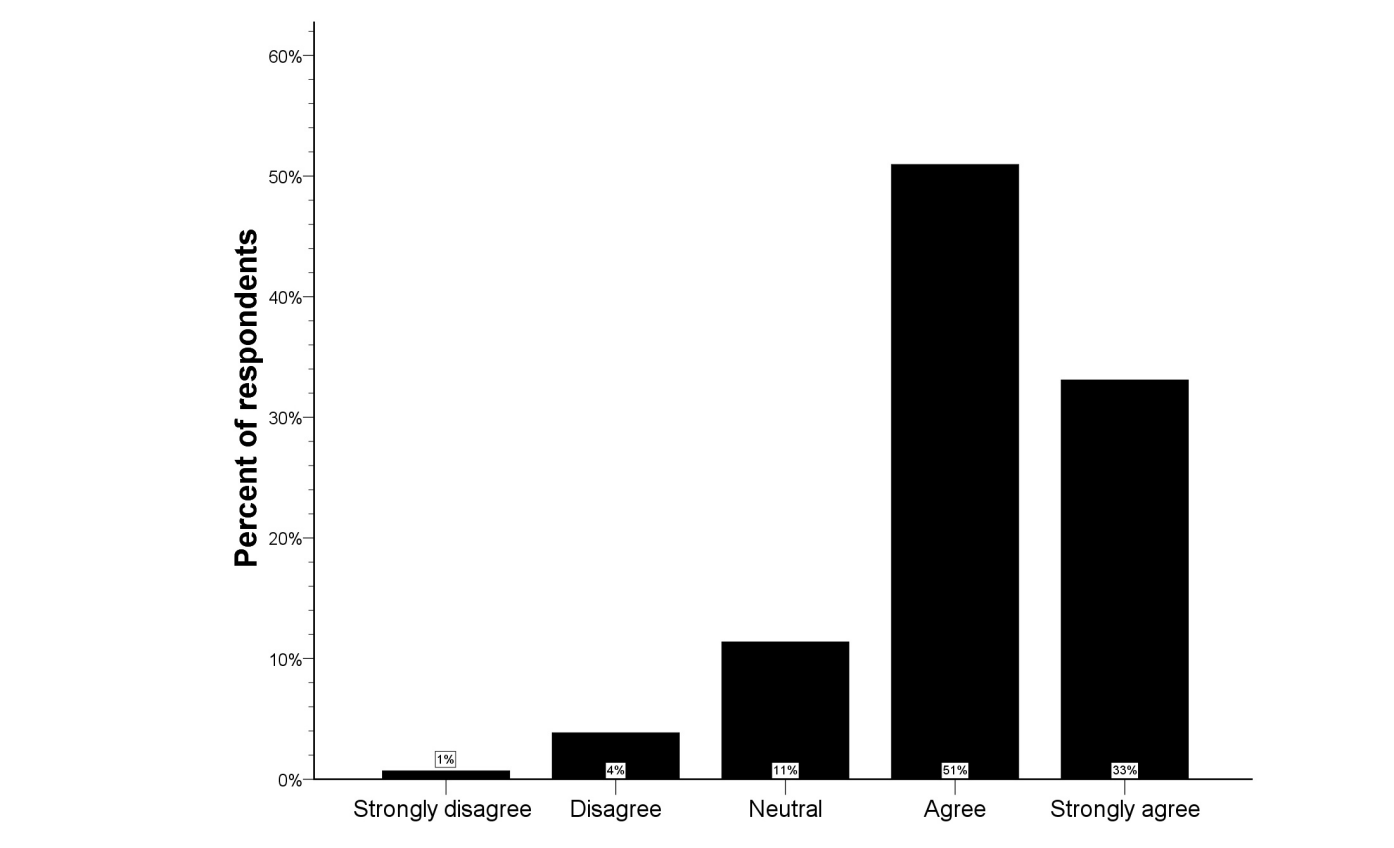
Distribution of the Likert scale for telemedicine at home in the total study population.

**Figure 5:**
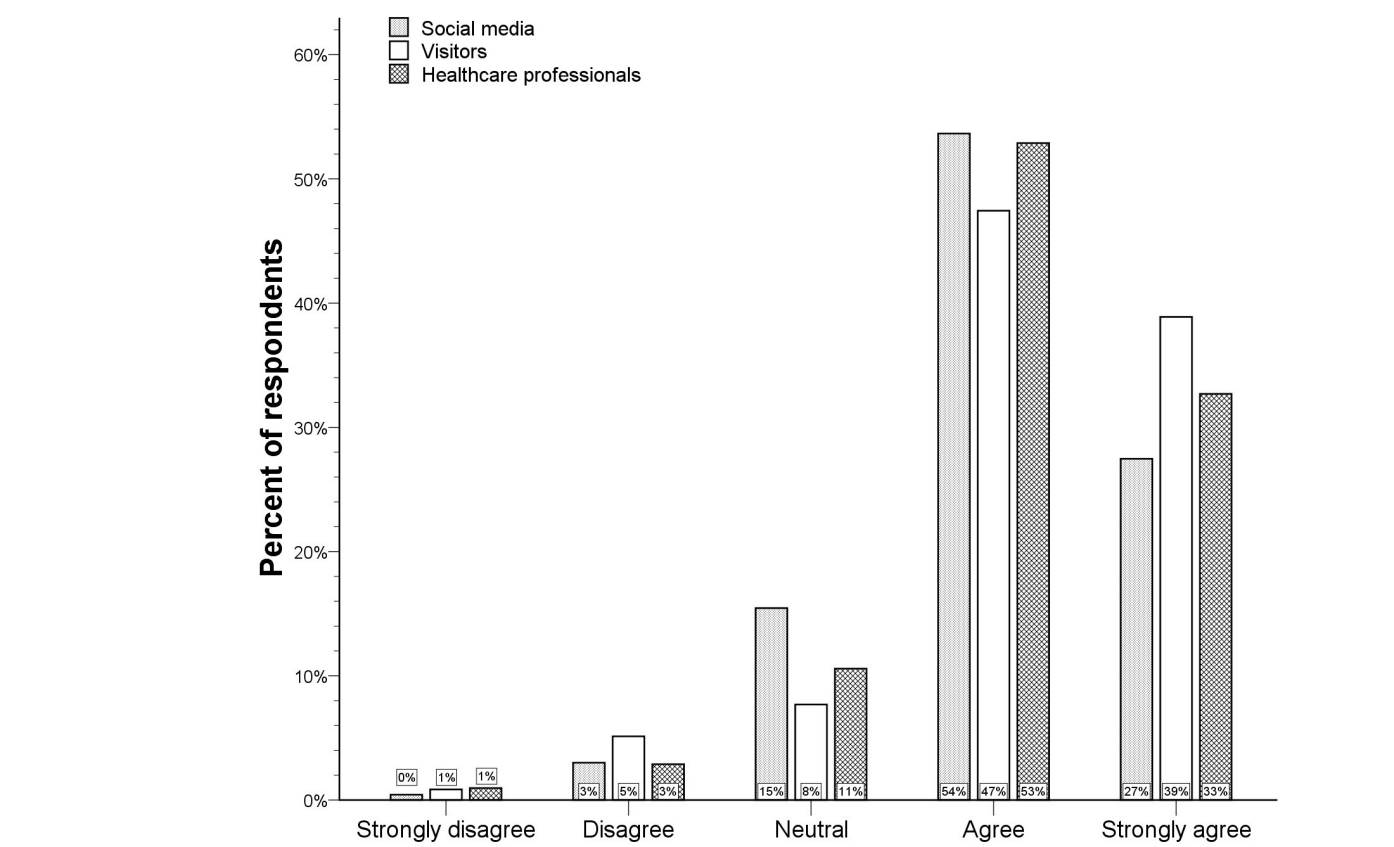
Distribution of the Likert scale for telemedicine at home per respondent type.

**Figure 6:**
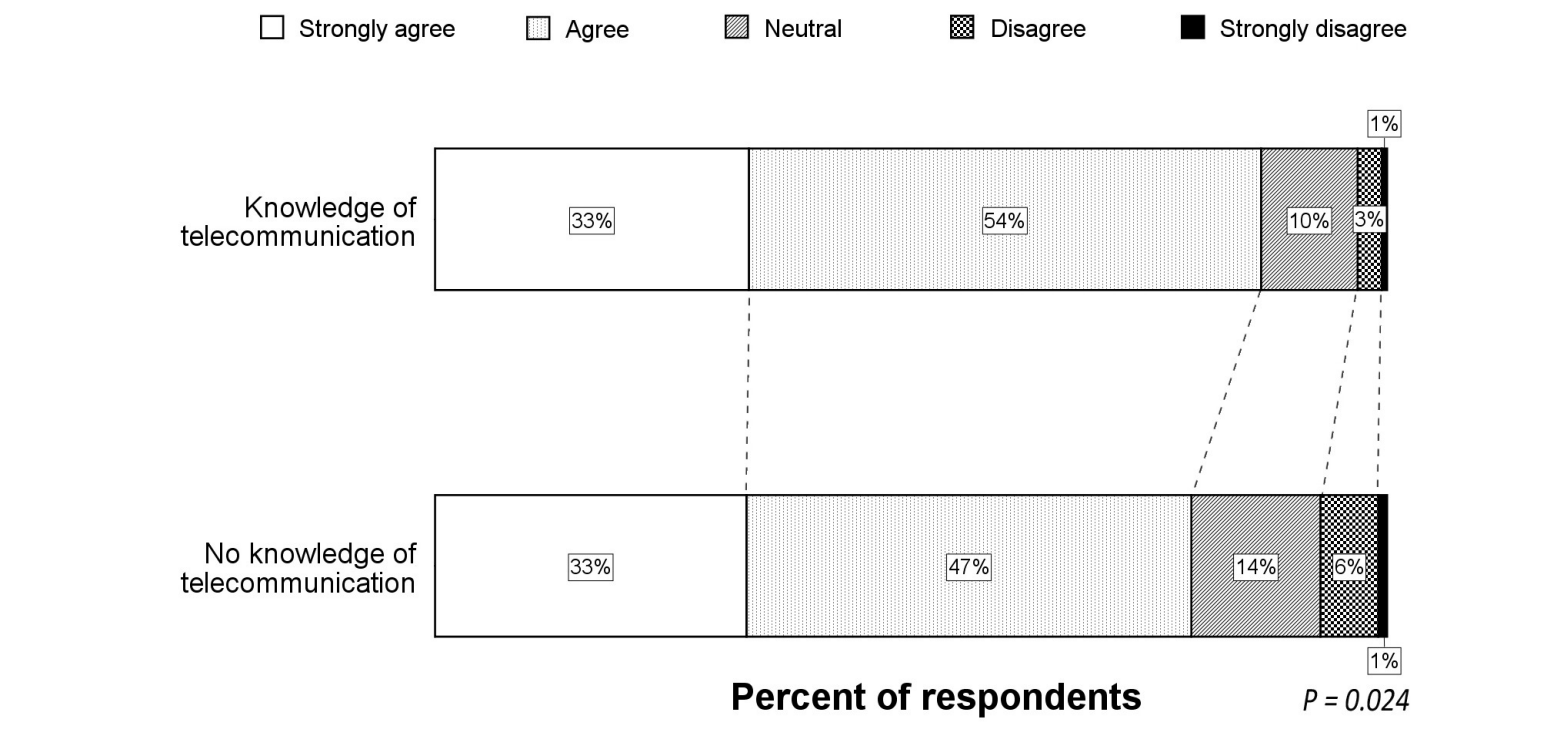
Shift analysis of the responses from respondents with knowledge of telecommunication compared to those without knowledge of telecommunication concerning telemedicine at home.

Figure 4 provides an overview of the opinions regarding the usefulness of telemedicine at home. Only 5% of all respondents was not convinced that telemedicine at home would be useful (median score 4, IQR 4-5). In univariate analysis, knowledge of computer systems for telecommunication was associated with more positive responses (*P=*.041), but there was no significant difference among the three respondent types (see Figure 5). Lack of telecommunication knowledge was the only independent predictor for rejection of telemedicine at home (logistic regression analysis; OR 0.36, 95% CI 0.16-0.83; *P=*.016), and there was a significant shift towards more positive answers in respondents with knowledge of telecommunication compared to those without telecommunication knowledge (*P=*.024) (see Figure 6).

### Protection of Privacy and Identity

**Figure 7:**
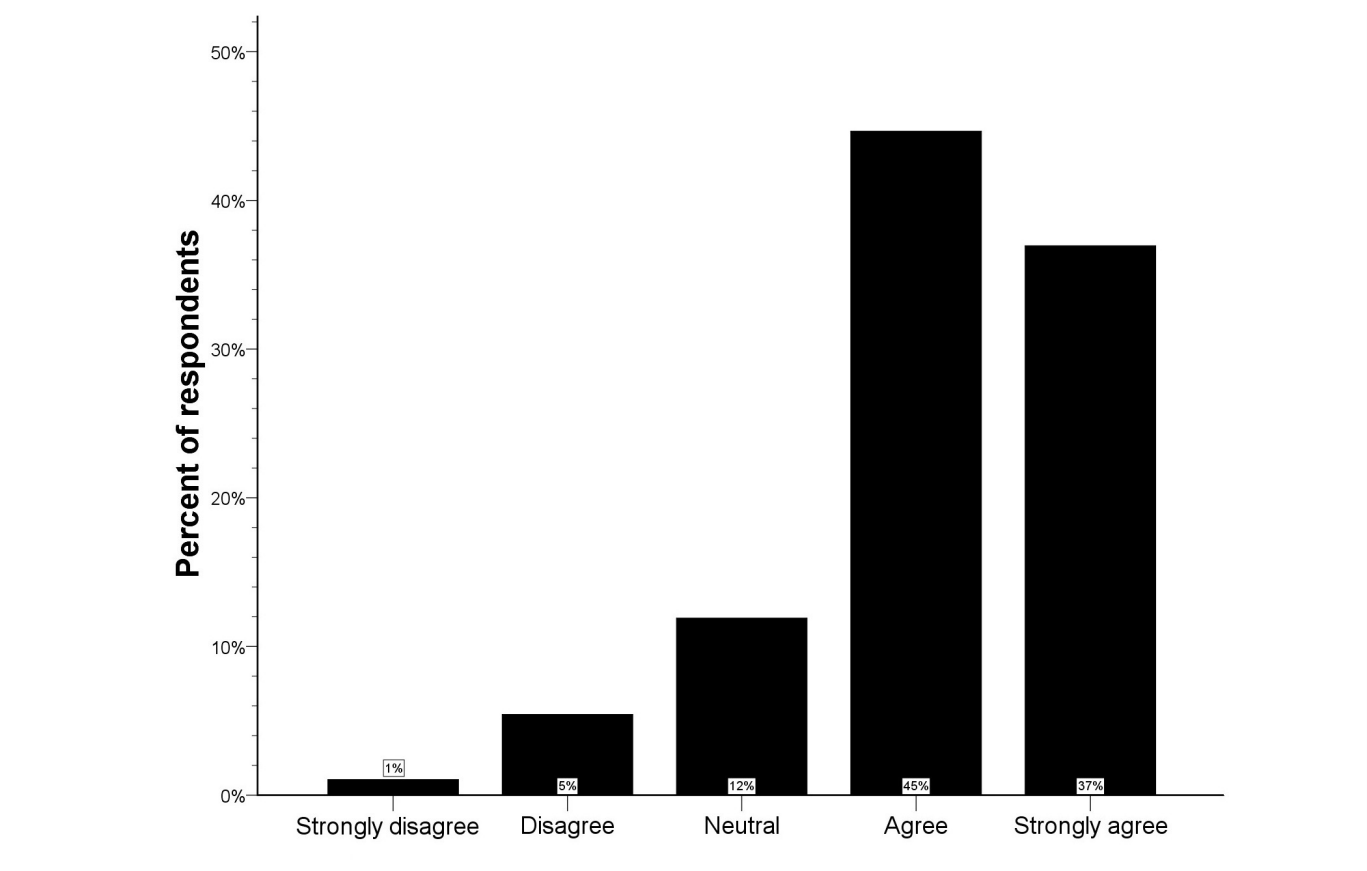
Distribution of the Likert scale for protection of privacy and identity in the total study population.

**Figure 8:**
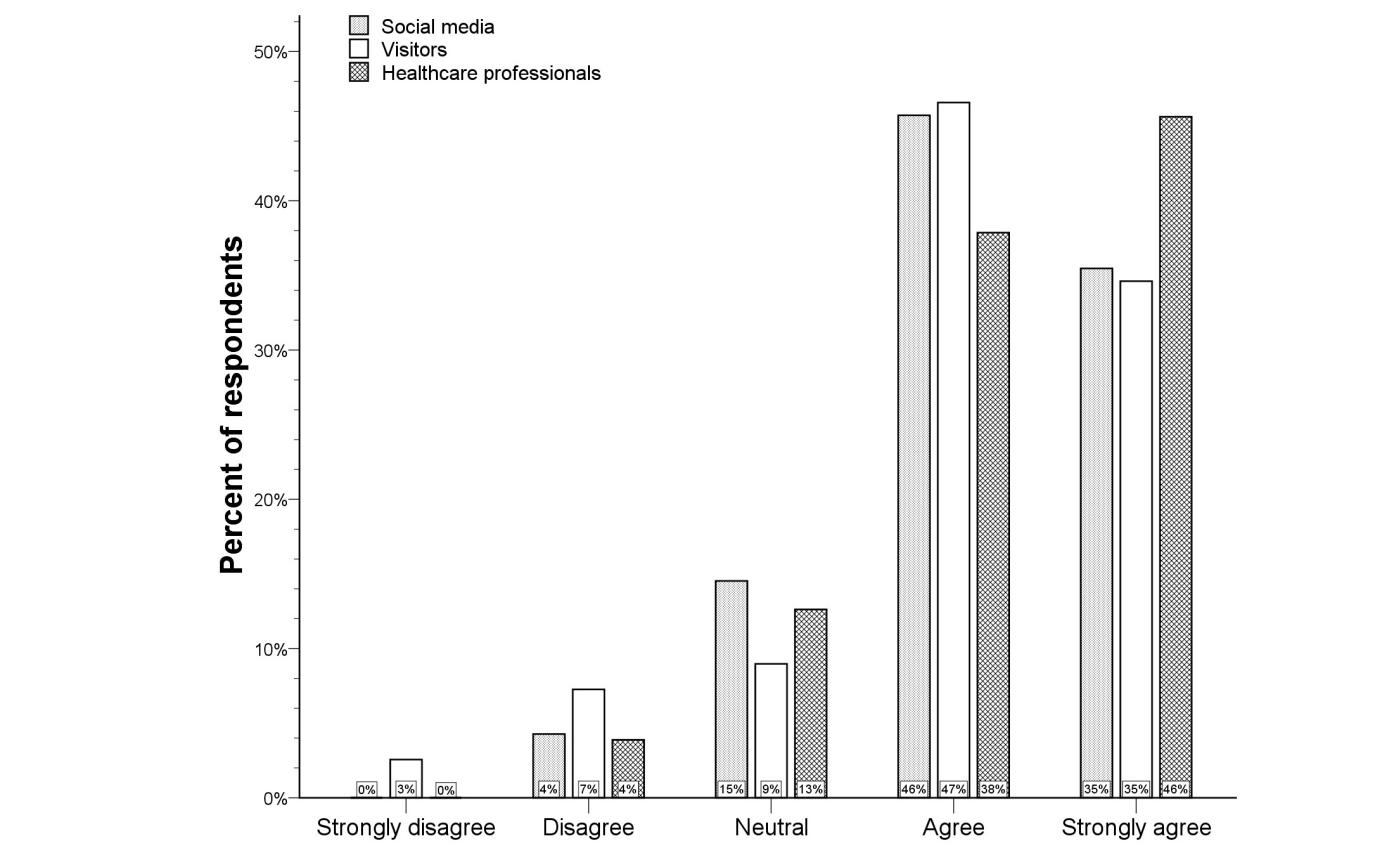
Distribution of the Likert scale for protection of privacy per respondent type.

**Figure 9:**
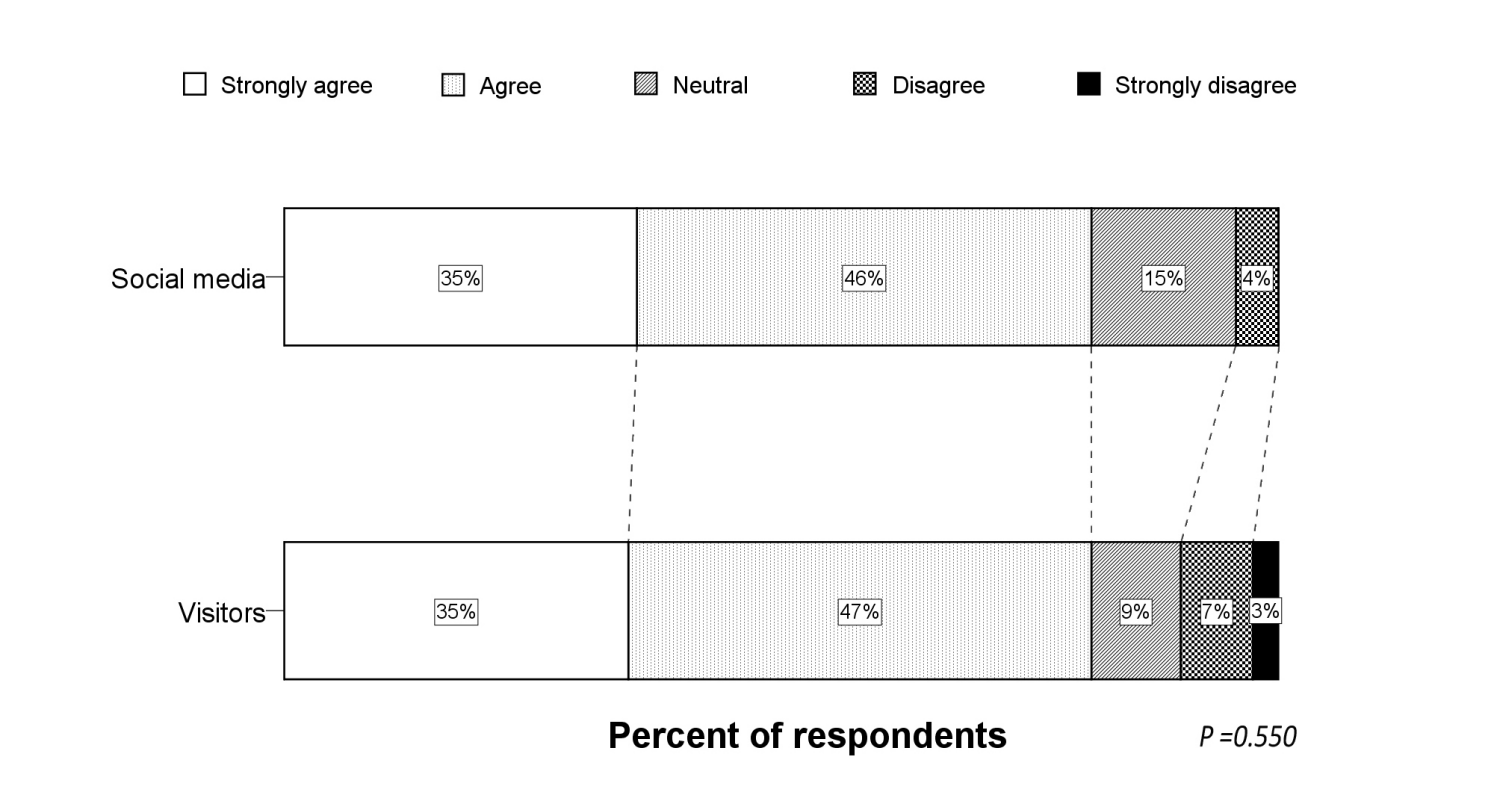
Shift analysis of the responses from social media compared to visitors concerning protection of privacy.

As show in Figure 7, only 7% of all respondents had no confidence that their privacy and identity would be protected during telemedicine consultations (median score 4, IQR 4-5). Univariate analysis indicated that respondents via social media were more concerned about privacy issues during telemedicine consultations than visitors (*P=*.033) (see Figure 8), which is a finding that was confirmed by logistic regression analysis (OR 0.44, 95% CI 0.20-0.95; *P=*.035). Shift analysis of the Likert scale showed that respondents via social media were more frequently neutral and less frequently disagreed or strongly disagreed than visitors (see Figure 9), but the shift over the entire spectrum was not statistically significant (*P=*.550).

### Future Participation in Telemedicine Consultations

**Figure 10:**
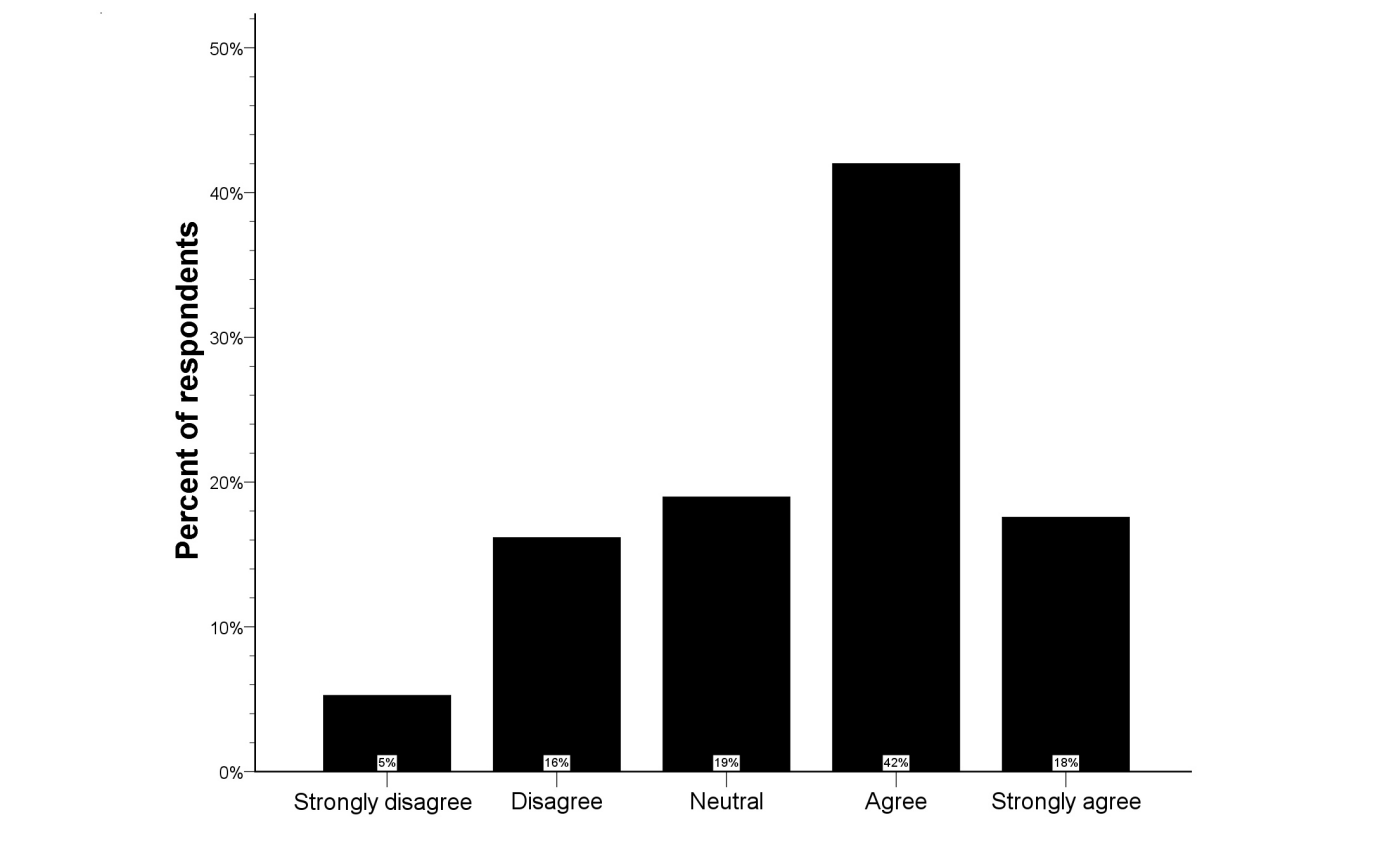
Distribution of the Likert scale for participation in future telemedicine consultations in the total study population.

**Figure 11:**
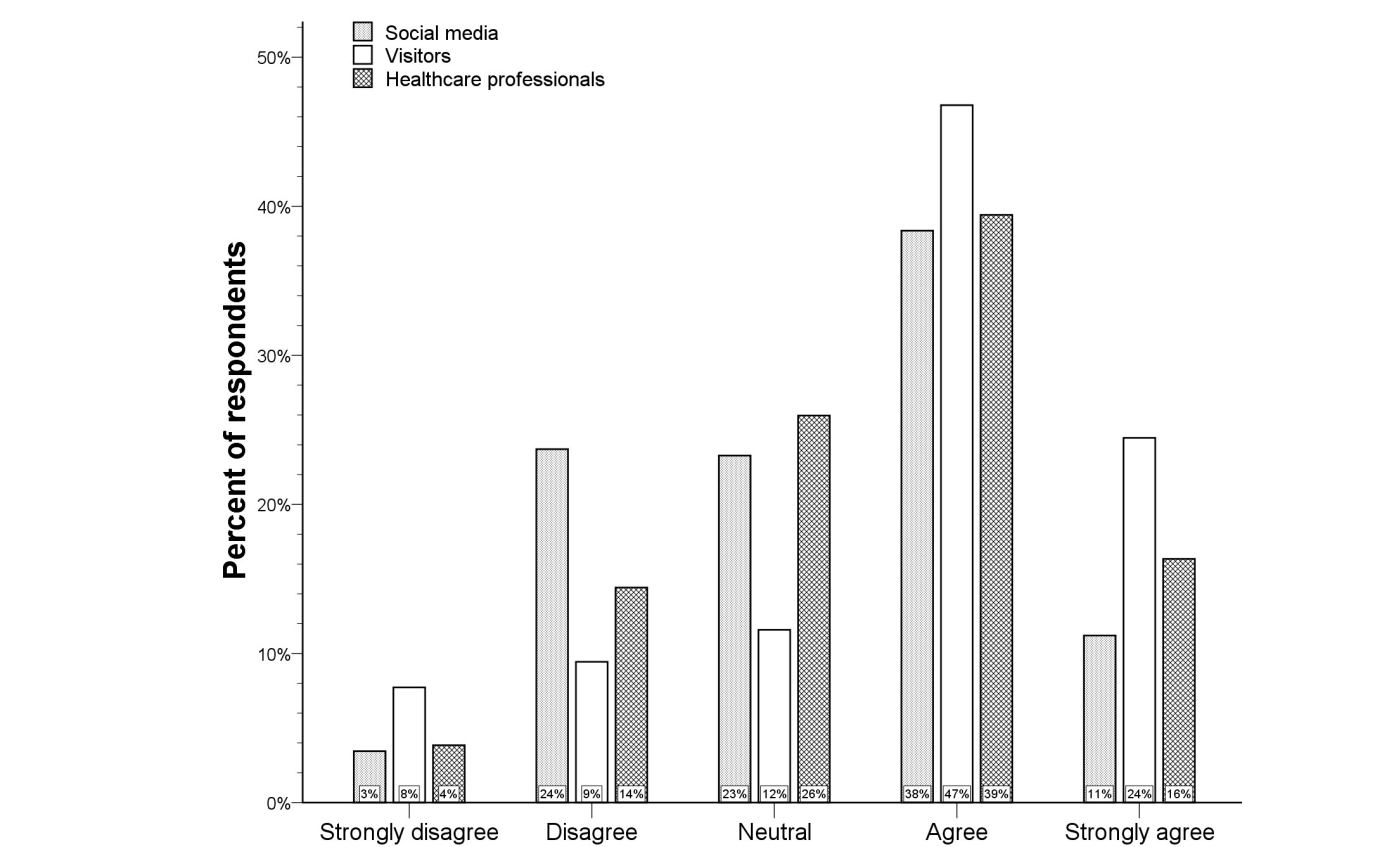
Distribution of the Likert scale for participation in future telemedicine consultations per respondent type.

**Figure 12:**
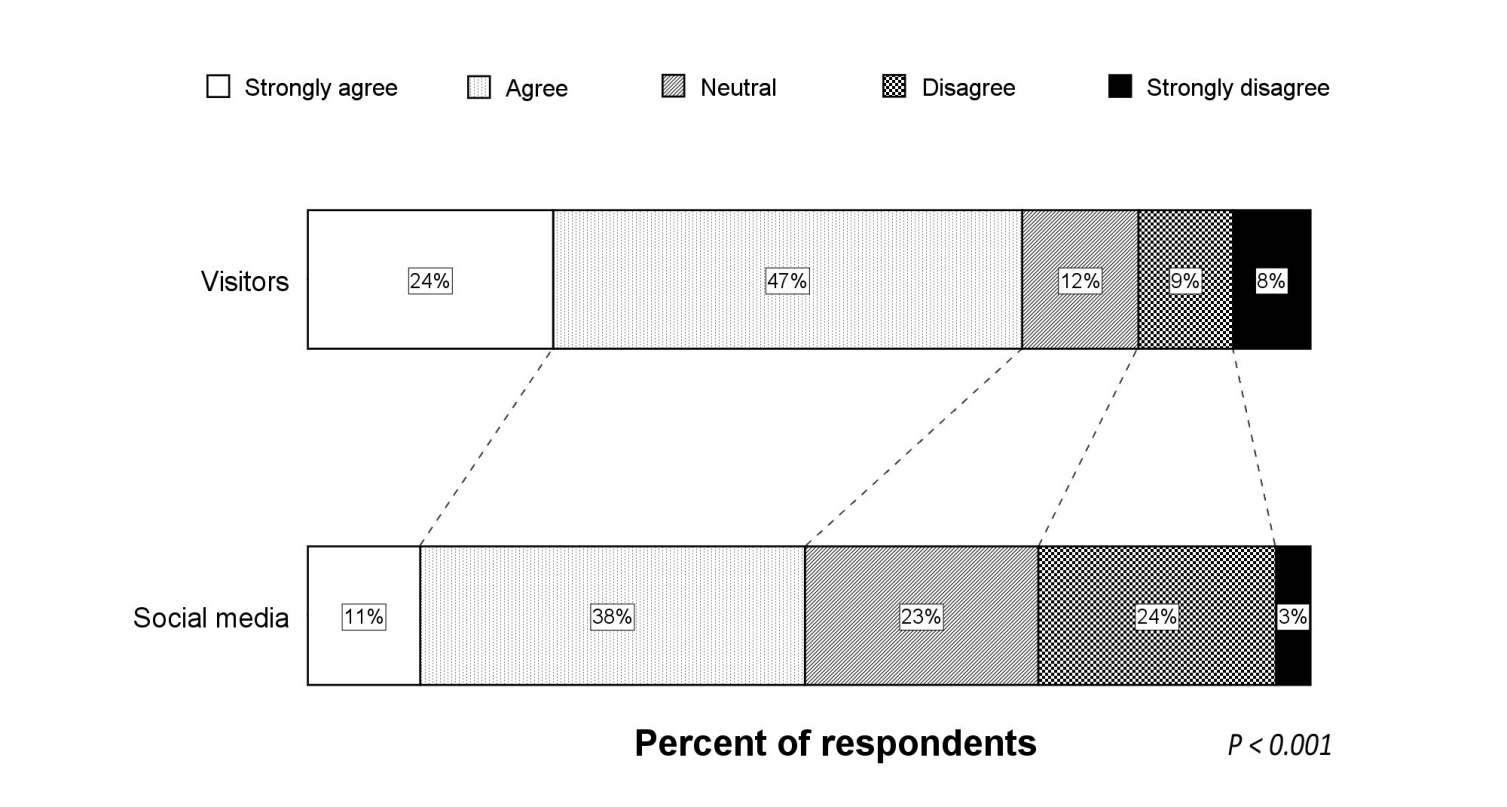
Shift analysis of the responses from visitors compared to social media concerning future participation in telemedicine consultations.

Most respondents indicated that they would agree to participate in future telemedicine consultations, but nearly a quarter of respondents disagreed or strongly disagreed (see Figure 10) (median score 4, IQR 3-4). Visitors were more likely to agree with future participation in telemedicine consultations than respondents via social media (*P*<.001) (see Figure 11). This association was confirmed by logistic regression analysis (OR 2.5, 95% CI 1.5-4.0; *P*<.001) and by shift analysis (*P*<.001) (see Figure 12).

### Composite Score

The median composite score was 16 (IQR 14-18; maximal 20), reflecting that the large majority of respondents expressed a positive overall attitude towards telemedicine. Older age was weakly correlated with higher scores (Spearman rho=.09; *P=*.038). Visitors (median 16, IQR 15-18) and professionals (median 16, IQR 14-18) provided more positive answers than respondents via social media (median 15, IQR 14-17; *P*<.001 and *P*=.025, respectively).

### Respondents’ Comments and Suggestions

Only 28 respondents (28/607, 4.6%) provided a comment in the last question. These comments can be categorized as (1) supportive of the further development of telemedicine (n=14), (2) additional information regarding the concept of telemedicine was needed for adequate completion of the survey (n=5), (3) telemedicine could be useful but nuances in patient-caregiver interaction may be lost (n=4), (4) concerns about technical aspects of telemedicine (n=3), and (5) telemedicine may be challenging for certain patient populations (eg, elderly, persons with autism spectrum disorder) (n=2).

### Construct of the Questionnaire

The internal consistency of the four Likert-scale questions was acceptable (Cronbach alpha=.66; 95% CI 0.62-0.71). Except for the item on protection of privacy during telemedicine consultations, all items contributed to the internal consistency. All four Likert-scale questions were intercorrelated (Spearman rho; *P*<.001 for all).

## Discussion

### Principal Results

The main finding of this study is the positive and congruent overall attitude regarding the implementation of telemedicine, both for in-ambulance emergency therapy and for chronic care at home. Privacy issues were not perceived as problematic, and most respondents were ready to participate in future teleconsultations.

### Comparison With Prior Work

Other surveys evaluating opinions about telemedicine services typically involve large cross-population inquiries [14] or report on the view of health care professionals [15] and specific patient populations [16]. To the best of our knowledge, we are the first to simultaneously present an identical questionnaire to the general public, professional caregivers, and stroke patients. This approach enables direct comparison of these key stakeholder views. It is especially noteworthy and reassuring that all three groups gave similar opinions about the application of telemedicine, the protection of privacy, and future participation in teleconsultations.

In contrast to prevailing prejudices and literature reports [17-19], older people appeared to be at least as eager to accept telemedicine in ambulances or at their homes as younger respondents. This finding is important as older patients make prime candidates for telemedicine given their increased risk of medical emergencies and higher need for long-term care.

More than one third of the study population had no knowledge of telecommunication technology. Interestingly, lack of telecommunication knowledge did not negatively impact the broad acceptance of telemedicine, except for teleconsultations at home. This may be explained by computer anxiety and the need to actively operate computer systems in the home care setting [11]. From the respondents’ perspective, this differs substantially from in-ambulance teleconsultations that are initiated and managed by a physician, allowing the patient to take on a more passive role.

The fact that respondents via social media less frequently expressed concerns with privacy and identity compared to visitors is another fascinating finding. The first group represents a younger population that is more familiar with information technology and teleconferencing. Their opinion is pertinent because they represent the potential future users of telemedicine, but whether their experience with social media warrants their optimism regarding protection of privacy and identity may be matter of debate.

### Strengths and Limitations

We deliberately designed a concise and user-friendly survey to limit the time needed for completion and to maximize the response rate. By doing so, we obtained a questionnaire with acceptable internal consistency that allowed us to collect the opinions of a substantial and representative study population. It should, however, be acknowledged that the small number of patients with a history of stroke hampers extrapolation of their results to the general stroke population. An inherent shortcoming of this survey is a possible selection bias as individuals with a negative or uninvolved stance towards telemedicine may have been less likely to participate, possibly resulting in overestimation of the positive general impression. Also, in contrast to respondents in face-to-face interviews, the concept of telemedicine was not clarified to those completing the survey online, nor did they have access to the prototype system for in-ambulance telemedicine. This discrepancy may be a cause of information bias and was also commented on by 5 respondents. Conversely, our study design allows the comparison of two survey data collection techniques, that is, the face-to-face interview and the use of an online questionnaire. The major strength of face-to-face surveys is the personal interaction and the possibility of providing additional clarification where needed, whereas online surveys allow inquiry of large numbers of respondents’ opinions rapidly and at little cost. Contrarily, respondents in face-to-face interviews are more susceptible to social desirability bias because of the interviewer’s presence, and the representativeness of online surveys may be questioned given their typical recruitment among younger individuals [20]. Specifically for this study, the availability of a prototype for in-ambulance telemedicine for participants in face-to-face interviews may have caused an additional bias [11]. For these reasons, the higher acceptance of in-ambulance telemedicine and the willingness for future participation in teleconsultations expressed by visitors participating in face-to-face interviews may not be surprising.

### Conclusion

The results of this survey indicate that the general public, professional caregivers, and stroke patients welcome telemedicine as a valid part of medical care for emergency treatment during ambulance transportation and for chronic care at home. Privacy concerns, older age, or lack of telecommunication knowledge were not identified as substantive roadblocks to implementation of these services.

## Abbreviations

IQR: interquartile range
UZB: Universitair Ziekenhuis Brussel
